# The Phantom Vanish Magic Trick: Investigating the Disappearance of a Non-existent Object in a Dynamic Scene

**DOI:** 10.3389/fpsyg.2016.00950

**Published:** 2016-07-21

**Authors:** Matthew L. Tompkins, Andy T. Woods, Anne M. Aimola Davies

**Affiliations:** ^1^Department of Experimental Psychology, University of OxfordOxford, UK; ^2^Crossmodal Research LaboratoryOxford, UK; ^3^The Australian National University, CanberraACT, Australia

**Keywords:** misdirection, illusion, amodal completion, modal completion, pantomime, ecological perception, inferential perception, expectation

## Abstract

Drawing inspiration from sleight-of-hand magic tricks, we developed an experimental paradigm to investigate whether magicians’ misdirection techniques could be used to induce the misperception of “phantom” objects. While previous experiments investigating sleight-of-hand magic tricks have focused on creating false assumptions about the movement of an object in a scene, our experiment investigated creating false assumptions about the presence of an object in a scene. Participants watched a sequence of silent videos depicting a magician performing with a single object. Following each video, participants were asked to write a description of the events in the video. In the final video, participants watched the Phantom Vanish Magic Trick, a novel magic trick developed for this experiment, in which the magician pantomimed the actions of presenting an object and then making it magically disappear. No object was presented during the final video. The silent videos precluded the use of false verbal suggestions, and participants were not asked leading questions about the objects. Nevertheless, 32% of participants reported having visual impressions of non-existent objects. These findings support an inferential model of perception, wherein top-down expectations can be manipulated by the magician to generate vivid illusory experiences, even in the absence of corresponding bottom-up information.

## Introduction

The performance of magic is based on practical and theoretical knowledge of psychology (see Gregory, 1982; Kuhn et al., 2008; Macknik et al., 2008; Rensink and Kuhn, 2015). Performance magic, particularly sleight-of-hand or “conjuring,” represents a rich resource for experimental psychologists. In particular, sleight-of-hand magic tricks provide a unique opportunity to investigate illusory perceptions of complex dynamic scenes. Magicians have spent millennia informally experimenting with perception, attention, and memory (e.g., Christopher and Christopher, 1973; Thomas et al., 2015), and theoretical writings on magic, dating back hundreds of years (e.g., Scot, 1584; Hodgson and Davey, 1887), anticipated recent scientific accounts of psychological phenomena.

**FIGURE 1:**
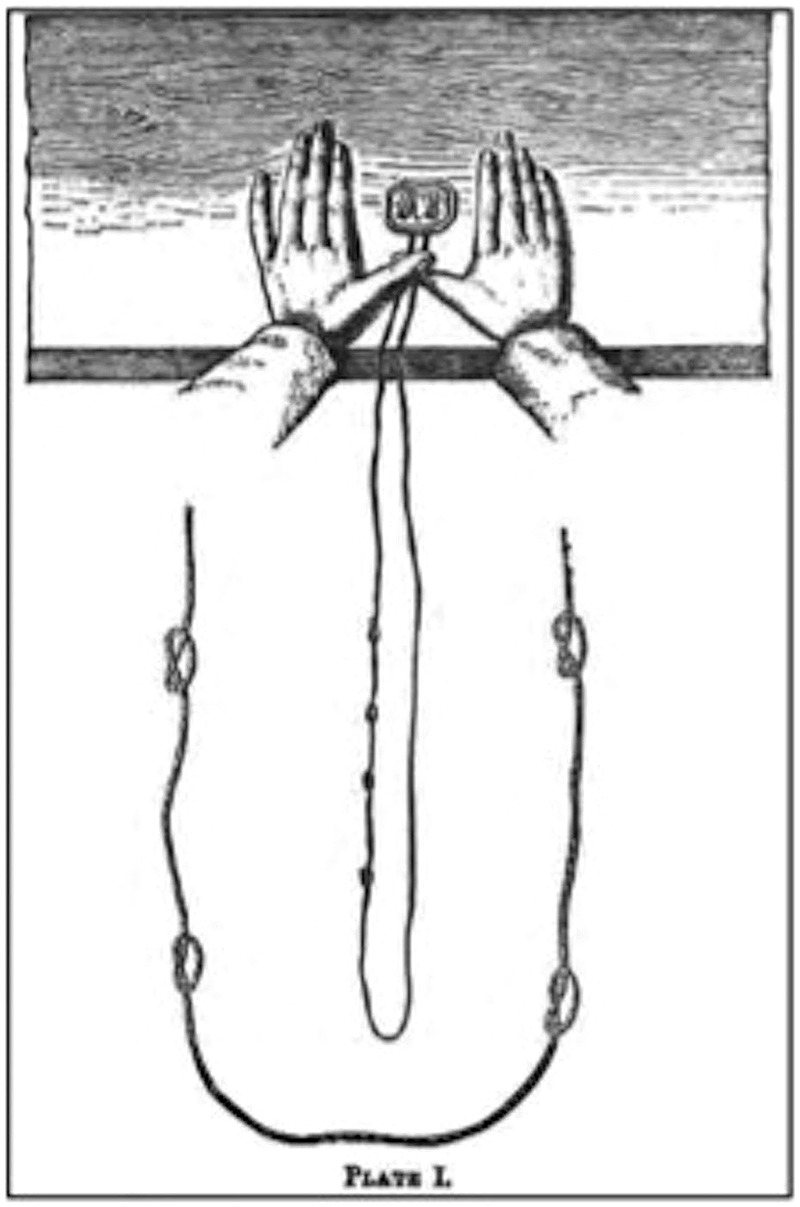
**Impossible knots in an endless cord (Zöllner, 1878).** Using this experiment, Professor Johann Zöllner described how a professional spirit medium could cause knots to form in a length of rope, even though the ends of the rope were sealed. Zöllner asserted that this was evidence of a supernatural power unexplained by modern science. Wundt (1879) argued that it was trickery. While Wundt was in the process of establishing the first Experimental Psychology Laboratory at the University of Liepzig, he became embroiled in a debate about the scientific value of investigating alleged supernatural phenomenon. His colleagues, including Johann Zöllner, Gustav Fechner and Ernst Weber, believed that they had discovered a new branch of “transcendental” physics, while Wundt maintained that they had been deceived by magic tricks or “jugglery.” This controversy gave rise to a series of articles by early experimental psychologists looking to investigate the relationship between illusory perception and beliefs (Jastrow, 1888, 1896; Dessoir, 1893; Binet, 1896; Triplett, 1900).

Empirical investigations of magic played a critical role in the establishment of Experimental Psychology as a scientific discipline (e.g., Wundt, 1879; see **Figure 1**), and early psychologists have written about the psychology of magic tricks (Jastrow, 1888, 1896; Dessoir, 1893; Binet, 1896; Triplett, 1900; see also Lamont, 2010; Thomas et al., 2016). However, performance magic was largely ignored by the scientific community throughout the twentieth century (Hyman, 1989). The first scientific study of magic to implement physiological measurements of adults perceiving magic effects was not conducted until 2005. Kuhn and Tatler (2005) used an eye-tracking paradigm to examine participants who watched a simple magic trick involving the apparent disappearance of a cigarette and a lighter. By integrating eye-tracking with sleight-of-hand-based stimuli, this experiment arguably marks one of the first scientific examinations of magic to move beyond the domain of observations, reviews, and opinion pieces into formal empirical investigation. This trend towards a “science of magic” has continued throughout the past decade, with researchers adapting magic tricks to investigate a wide variety of cognitive mechanisms. The present study builds upon previous research by introducing a novel paradigm designed to test how magicians can manipulate the way spectators perceive objects in dynamic scenes. While previous studies (e.g., Kuhn and Land, 2006; Beth and Ekroll, 2015) have demonstrated that magic tricks can cause spectators to make false assumptions about the movement of objects in a scene, the current study takes this a step further by testing whether misdirection can cause spectators to make false assumptions about the presence of objects in a scene.

Magicians have written extensively about the theory and practice of magic (e.g., Houdin, 1868/1881; Maskelyne and Devant, 1911), and it is useful to adopt some of their informal terminology when describing empirical investigations involving magic (e.g., Lamont and Wiseman, 1999). In this terminology, a “trick” consists of both an “effect” and a “method,” effect referring to the subjective experience of the spectators, and method referring to the mechanisms by which the effect is achieved (see Lamont, 2015; Rensink and Kuhn, 2015 for a discussion of classifying magic tricks based on methods and effects). For a trick to be successful, the performer must disguise the true method behind the effect, creating an “illusion of impossibility” (e.g., Nelms, 1969; Ortiz, 2006); the manipulations used to accomplish this are referred to as “misdirection.”

Misdirection is a particularly elusive term (e.g., Lamont and Wiseman, 1999; Kuhn et al., 2014). To date, most psychological considerations of misdirection have focused almost exclusively on how misdirection can be used to conceal objects and events from spectators (e.g., Lamont and Wiseman, 1999; Kuhn and Findlay, 2010; Memmert, 2010). Existing paradigms tend to focus on how to prevent spectators from detecting ostensibly visible elements of the methods behind magic effects. These failures to see have been associated with phenomena such as inattentional blindness (Kuhn and Tatler, 2005; Barnhart and Goldinger, 2014) and change blindness (e.g., Johansson et al., 2005; Smith et al., 2012, 2013). But misdirection does not only involve inducing failures to see, it can also involve inducing misperceptions of illusory objects. The one notable exception to this trend of focusing on concealment is the empirical investigation of the Vanishing Ball Illusion. This effect was first introduced into the psychological literature by Dessoir (1893), an early psychologist and amateur magician (Whaley, 2006). Dessoir described how a magician might induce the misperception of an illusory object – by tossing an orange into the air two times, then secretly dropping the orange into his pocket while pantomiming a third toss with his empty hand. Spectators would misperceive the orange leaving the magician’s hand and disappearing into the air on the third toss.

Triplett (1900) conducted actual informal experiments with schoolchildren, in which he performed a similar trick using a tennis ball. About half the children reported that they had perceived the ball rise towards the ceiling and then vanish. This Vanishing Ball Illusion has been adapted by Kuhn and Land (2006), who demonstrated that 63% of adult observers reported an illusory ball. They also argued that eye-tracking recordings suggested that social cues from the magician contributed to the illusion, that is, spectators who experienced the Vanishing Ball Illusion (the misperception of an illusory ball) looked to the magician’s eyes and were misdirected by his gaze as he looked upwards during the false throw. Subsequent studies have demonstrated that this magical effect remains relatively robust even without deceptive social cues (Thomas and Didierjean, 2016a; for a broader discussion of the role of social cues in magic see Cui et al., 2011; Tachibana and Kawabata, 2014; Kuhn et al., 2016). More recent research has demonstrated that the illusion can also be induced even in the absence of the initial “real” throws (Kuhn and Rensink, 2016).

Other studies involving sleight-of-hand magic tricks have used the false transfer method to examine the degree of “magicalness” of performances. Beth and Ekroll (2015) showed participants a series of videos of a magician performing magic tricks that included several “vanishes.” The effect was that a poker chip seemed to disappear inexplicably, and this was accomplished with a method known as a false transfer – the magician pretended to pass a poker chip from one hand to his other, while secretly retaining it in his first hand. By manipulating the timing between the moment of the false transfer and the revelation that the poker chip was not in the magician’s hand, they found that participants would rate the quicker revelations of the empty hand as being relatively more magical. The authors suggested that such vanishing effects could be linked with the ideas of modal and amodal completion – perceptual experiences that are not directly drawn from any sensory modality (see also Nanay, 2009; Barnhart, 2010; Ekroll et al., 2013).

While many studies of sleight-of-hand magic tricks have focused on the role of spectators’ perceptions, an additional small body of literature focuses specifically on the physical actions of the magician’s hands. For example, one study (Cavina-Pratesi et al., 2011) has demonstrated that practicing magicians are significantly more skillful at pantomiming actions compared to control participants (non-magicians). When asked to pantomime the action of picking up an object, control participants made hand motions that were notably different from genuine grasping gestures. In contrast, the fake grasping gestures of the magicians were more kinematically similar to their genuine grasping actions. Such expertise contributes to the deceptiveness of sleight-of-hand performances (Phillips et al., 2015), and surveys of professional magicians indicate that they place a particularly high value on pantomimic expertise (e.g., Rissanen et al., 2014).

The present study extends previous research on the false transfer method and the Vanishing Ball Illusion by introducing a novel magic trick, adapted by the first author. The Phantom Vanish Trick was created to investigate the idea that participants can form vivid illusory impressions of objects in response to magic performances. The method is inspired by a sleight-of-hand technique historically referred to as a “bluff vanish” (e.g., Shephard, 1946; Bobo, 1952). In the original method, the magician begins by clearly and openly showing the spectators that he is holding a handful of mixed coins. Then, with his other empty hand, he reaches into the handful of coins and pantomimes the action of taking away a single coin. The magician does not actually take anything from the handful of coins, but he does (falsely) verbally indicate to the spectators that he has taken one of the coins. Next, the magician disposes of the “remaining” coins into his pocket (really *all* of the coins go into the pocket, since he did not actually take any coin away from the original handful). Finally, the magician goes through the pantomime of making the single coin disappear. This trick is effectively a false transfer that depends both on the convincingness of the pantomime and also on the spectator not being able to count the original handful of coins. The Phantom Vanish Trick streamlines this idea by eliminating the handful of coins altogether. The magician simply pantomimes the actions of presenting an object and making it disappear. A real object is never presented at any point during the trick. Additionally, in the current experiment, the Phantom Vanish Trick was presented in the context of a silent video, meaning that the magician was not able to use false verbal information to mislead the spectators.

The Phantom Vanish Trick represents a novel contribution to the perception literature in that it has the potential to demonstrate that a spectator’s top-down expectations can lead them to perceive illusory objects where none have been presented. This is an extension of previous experiments that have shown that people may falsely infer the illusory motion of an object. For example, in the Vanishing Ball Illusion, spectators reported seeing an illusory ball leave the magician’s hand. Similarly, Cui et al. (2011) reported that participants falsely perceived a coin being tossed by a magician from one hand to the other, despite the fact that the coin was actually retained in the initial hand that was making the toss.

Proponents of ecological theories of perception have made strong predictions about the potential for healthy adults to misperceive objects. Gibson (1982) asserted that it is impossible to induce the false visual perception of an object where none exists (barring optical illusions or pharmacological or psychiatric considerations). He states:

“Do we ever really “see” a non-existent object or place as if it existed? I do not mean the *virtual* object in a mirror, or a *pictured* object behind the picture, or a *mirage* in the desert air, but a *hallucinated* object, a thing for which no invariants are present in the ambient light even when the presumably drugged or diseased observer walks around it. If it is true that the absence of all structure in the light specifies air, i.e., “nothing” in the sense of *no thing*, the answer must be that we do not and cannot (p. 223, original emphasis).”

While ecological theorists assert that human phenomeno logical experience is derived directly from bottom-up sensory information, inferential theorists (e.g., Helmholtz, 1867; Gregory, 1997, 2009) propose that phenomenological experiences are derived from top-down interpretations of bottom-up sensory information. Thus, if participants do report the presence of objects after viewing the Phantom Vanish Trick, this would support an inferential theory of human visual perception. Such reports would imply that top-down information, in this case, the strong expectation that the object is present, is subjectively indistinguishable from veridical sensory information. In other words, participants will have the experience of seeing an object even though it is not presented because they think that it ought to be there.

Based on informal observations of professional magic trick performances, as well as previous studies of sleight-of-hand magic tricks and pantomimes (e.g., Kuhn and Land, 2006; Phillips et al., 2015), we predicted that some participants who watched the video of the Phantom Vanish Trick would report the presence of a non-existent object, and that there were three possible outcomes. Of the participants who did experience the Phantom Vanish Illusion (PVI), some would indicate that they saw the magician make “something” disappear while others would indicate that they saw the magician make a specific object disappear (e.g., a “silver coin” or “red ball”). The third possible outcome was that some participants would fail to experience the PVI, and they would simply provide a veridical report of the events shown in the video.

## Materials and Methods

### Participants

Participants were recruited to take part in the study online (see Woods et al., 2015 for a review of online behavioral research methods) through Amazon’s Mechanical Turk.^1^ There were 420 participants who completed the study (mean age = 33.5 years; age range = 19–73 years; male = 237), and an additional 23 participants who were excluded from the analysis because they did not complete the experiment. All participants self-reported as having normal or corrected-to-normal vision and no history of neurological illness or injury. Participants were tested following a protocol approved by the University of Oxford Research Ethics Committee, and in accordance with the ethical standards laid down in the 2008 Declaration of Helsinki. Each participant completed the experiment individually online and was given US $1.50 as compensation for their time.

### Stimuli and Procedure

The study was conducted online using Adobe Flash-based Xperiment software.^2^ Participants completed the experiment using their own computers, and at the start of the study, participants had the option of viewing the stimuli in a discrete browser window or in “full-screen” mode.

**FIGURE 2:**
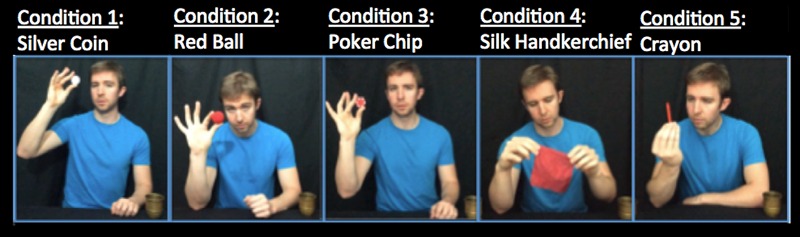
**Five different object conditions were used in the experiment.** In the first four videos of the five-video sequence, participants only ever saw one of the five objects – silver coin, red ball, poker chip, silk handkerchief, or crayon. In Video 5 there was no object presented.

Stimuli consisted of a total of 22 videos. All videos were recorded in 1080 HD, at 30 FPS, using an iPhone 5S, and edited for length in iMovie. All of the videos were silent, to control for the fact that participants would be watching on their personal devices with varying audio capabilities. The stimuli set included one “practice” video, and one “critical” video – the Phantom Vanish Trick. There was only one version of each of these two videos, and they were shown to every participant. The other 20 videos included 15 “magic trick” videos and five “non-magic control” videos. There were three types of magic trick videos: Video 1, Miscellaneous Trick; Video 2, Vanish Trick; Video 4, Appearance Trick, and one type of control video: Video 3, Non-Magic Control. There were 20 videos because each of these four types of videos (Miscellaneous, Vanish, Appearance, and Non-Magic Control) was performed with five different objects: Condition 1, Silver Coin; Condition 2, Red Ball; Condition 3, Poker Chip; Condition 4, Silk Handkerchief; Condition 5, Crayon. See **Table 1** for the number of participants in each of the five object conditions, and **Figure 2** for an illustration of the five different object conditions.

**Table 1:** Number of participants in each of five different object conditions.

Condition	Object	Participants
1	Silver coin	81
2	Red ball	80
3	Poker chip	100
4	Silk handkerchief	79
5	Crayon	80

**FIGURE 3:**
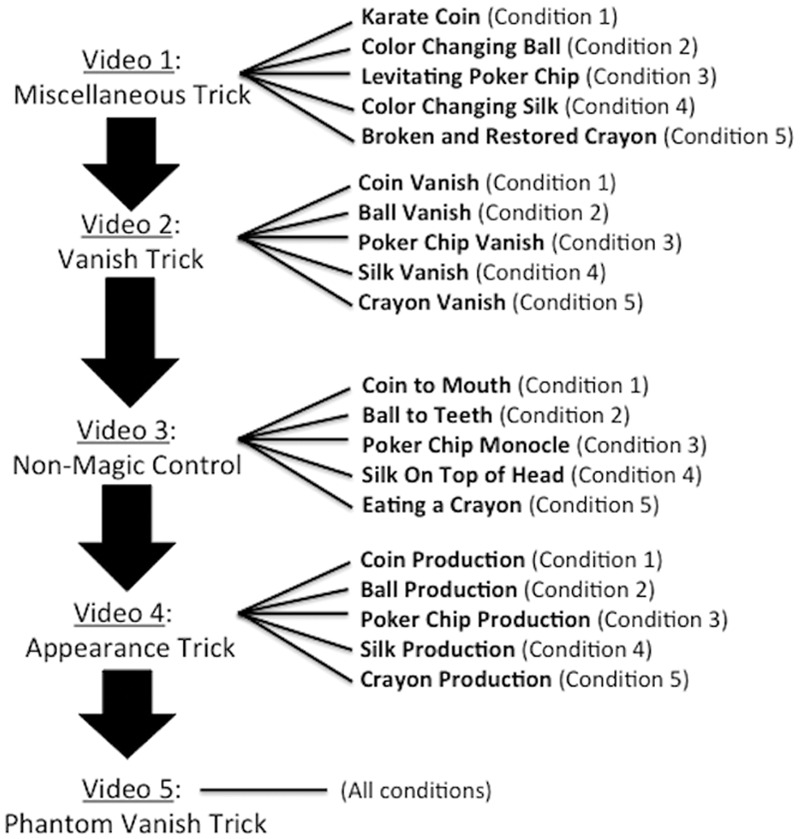
**Participants were presented with a five-video sequence – Video 1: Miscellaneous Trick; Video 2: Vanish Trick; Video 3: Non-Magic Control; Video 4: Appearance Trick; Video 5: Phantom Vanish Trick.** The first four videos depicted a magician performing with a single object – either a silver coin (Condition 1), a red ball (Condition 2), a poker chip (Condition 3), a silk handkerchief (Condition 4), or a crayon (Condition 5). The object varied for participants, so that one group of participants watched a five-video sequence involving a silver coin (Condition 1) while another watched a five-video sequence involving a red ball (Condition 2), etc. The order of the tricks in the five videos that constituted a video sequence was intended to approximate a routine from a magic show.

**FIGURE 4:**
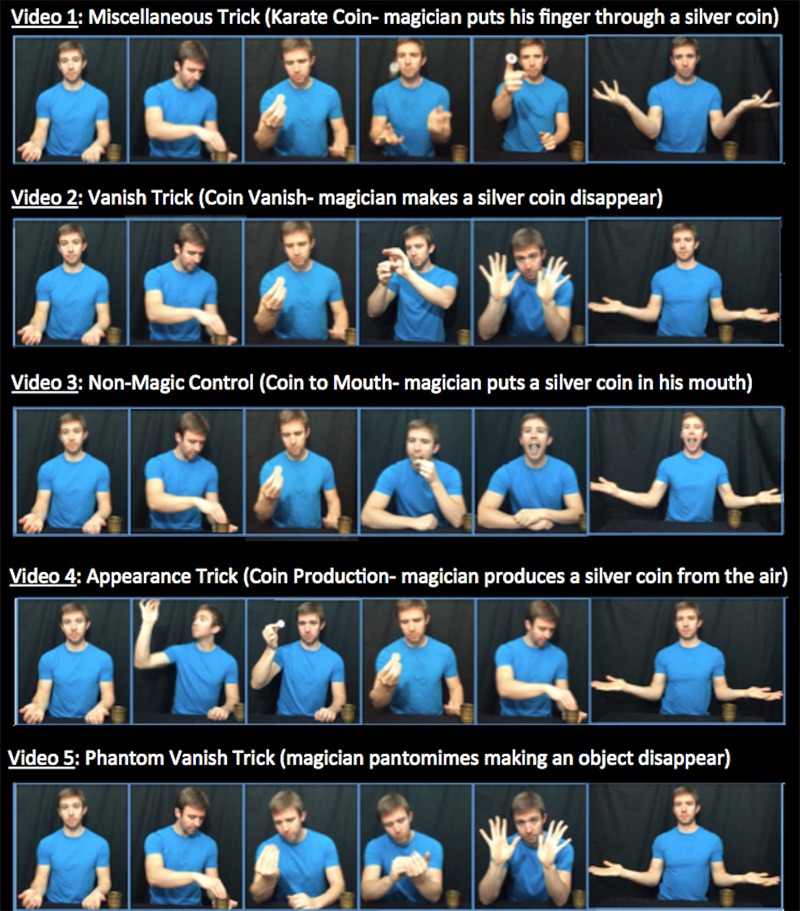
**An illustration of a five-video sequence, as viewed by a participant.** All participants in Object Condition 1, Silver Coin, watched Videos 1–4 (Miscellaneous Trick, Vanish Trick, Non-Magic Control, Appearance Trick) that depicted a magician performing with a silver coin before they watched Video 5 (Phantom Vanish Trick), which did not include an object. Note that participants in Object Conditions 2–5 also watched similar five-video sequences involving different objects. Regardless of which object condition the participants were in for Videos 1–4, the Phantom Vanish Trick (Video 5) was identical for every participant.

Participants watched a five-video sequence that was presented in an order designed to approximate a routine that might be performed within the context of a magic show. See **Figure 3** for a breakdown of the five-video sequences that were possible with each of the five different object conditions. In all of the videos, a brass cup was visible on the table to the left of the magician. The cup was a receptacle for the objects. The first four videos in the sequence (which always showed an object) were intended to establish an expectation that the magician would take an object out of the cup, while the fifth video (which did not show an object) served as the critical video. See **Figure 4** for an illustration of a five-video sequence. The complete set of videos can be viewed online^3^.

Videos 1, 2, and 4 were presented as magic tricks. They were designed to establish that the magician was performing magical actions with the object. The tricks were presented so that the methods could not be easily inferred from the video, assuming that the participant did not have prior knowledge of the methods behind magic tricks. Video 1, the Miscellaneous Trick, showed the magician doing something magical with the object (e.g., breaking it and magically restoring it, or magically changing its color). Video 2, the Vanish Trick, showed the magician making the object seemingly disappear. Video 4, the Appearance Trick, showed the magician apparently producing the object from thin air.

Video 3, the Non-Magic Control, served as a manipulation check for demand characteristics. Participants had been informed that they would be watching a series of magic tricks, which might have led them to describe magic tricks even when the video did not depict a magic trick. Video 3 did not depict any apparent magical or impossible events (e.g., Video 3, Object Condition 1 depicted the magician placing the silver coin between his teeth). Therefore, if participants did report seeing magical or impossible events after watching this video, we would be unable to rule-out the influence of demand characteristics on participants’ responses to Video 5, the Phantom Vanish Trick.

Video 5, the Phantom Vanish Trick, served as the critical video of the experiment. Participants’ responses to this video directly addressed our central question: Could a silent pantomime of a magic trick result in reports of objects where none were presented? This video showed the magician pantomiming the action of removing an object from the cup and then going through the motions of making the non-existent object disappear. Unlike the first four videos, no object was shown in the Phantom Vanish Trick.

Participants were asked to write a description of each video (Question 1) and to provide three ratings of how surprising (Question 2), how impossible (Question 3), and how magical (Question 4) they found the video. At the end of the experiment, after watching all of the videos, participants were asked to report how interesting they generally considered magic tricks to be (Question 5). See **Table 2**, the *Spectators’ Experience Questionnaire*, for the complete list of questions. The ratings for Questions 2–4 were collected using a series of visual analog scales. Participants were presented with a continuous line anchored at one end with the words “not at all surprising” (or impossible or magical) and at the other end with “very surprising” (or impossible or magical). For each rating (of surprising, impossible or magical), participants were instructed: “Please use your mouse to indicate your response on the slider below” (see Reips and Funke, 2008 for a discussion of using computer-based visual analog scales).

**Table 2:** Spectators’ experience questionnaire.

	Response format	Question
Q1	Written Verbal Response	Please write a description of what was shown in the video. Do your best to describe specific actions and events in the order that they occurred.
Q2	Visual Analog Scale (from “not at all surprising” to “very surprising”)	How surprising did you find the events shown in this video?
Q3	Visual Analog Scale (from “not at all impossible” to “very impossible”)	To what degree did the events shown in this video seem to be physically impossible?
Q4	Visual Analog Scale (from “not at all magical” to “very magical”)	How magical did you find the events shown in this video?
Q5^∗^	Visual Analog Scale (from “not at all interesting” to “very interesting”)	In general, how interesting do you consider magic tricks to be?

*^∗^Participants answered Q1–Q4 a total of five times, that is, once after each video in the five-video sequence, but they answered Q5 only once at the end of the experiment.*

The critical question was Question 1 for Video 5 (Phantom Vanish Trick). The participants’ responses to this question allowed us to determine whether they had experienced the PVI. The ratings for Questions 2–4 for Video 5 were intended to corroborate the written reports (i.e., participants who experienced the PVI should consider Video 5 to be more magical and/or impossible than those who did not experience the illusion). Throughout the experiment, the questions served to keep the participants actively engaged with the videos, and by asking the same questions about every video in the sequence, we avoided placing any special emphasis on Video 5 (Phantom Vanish Trick) that might have otherwise influenced the participants’ responses.

In summary, the experiment began with the participants being informed, through onscreen written instructions, that they would be watching a series of short (less than 30 s) videos. They were told that they would be able to control when the videos started and that, during the experiment, each video could only be played once. Participants then completed the practice trial, and they were given the option to repeat the practice trial or to begin the experiment. The practice trial included a video, depicting the magician magically transforming one playing card into another, followed by Questions 1–4. Once participants confirmed that they wished to begin the trial, they were presented with a written cue: “Press SPACE to start the trial.” Pressing the spacebar initiated the trial. The practice trial was in an identical format to the experimental trials; that is, after each video ended, participants were presented with Questions 1–4 of the Spectators’ Experience Questionnaire (see **Table 2**). For each experimental trial, participants were required to answer each question (by typing text for Question 1 and by clicking on the visual analog scale slider for Questions 2–4) before they watched the next video in the sequence. This process was repeated until participants had watched all five videos in the five-video sequence and responded to the four questions following each video. The *five* five-video sequences differed by the object that was used in Videos 1–4, but Video 5, the Phantom Vanish Trick, was the same for all participants regardless of which object condition they participated in. Finally, every participant answered one additional question (Question 5 of the Spectators’ Experience Questionnaire): “In general, how interesting do you consider magic tricks to be? Please use your mouse to indicate your response on the slider below.” Participants indicated their responses by clicking with their mouse at a point along a continuous line anchored at one end with the words “not at all interesting” and at the other end with “very interesting.”

## Results

### Participants’ Written Reports for Question 1 on the Spectators’ Experience Questionnaire

Question 1 (Q1) of the Spectators’ Experience Questionnaire was presented immediately after each individual video of the five-video sequence, and the participants were asked:

“Please write a description of what was shown in the video. Do your best to describe specific actions and events in the order that they occurred.”

#### Participants’ Written Reports for the Magic Tricks (Videos 1, 2, and 4)

Videos 1, 2, and 4 were designed to be perceived as conventional magic tricks; each video depicted a trick that involved a single effect intended to create an apparent illusion of impossibility. As predicted, participants reported that they found the videos to be both impossible and magical. Overall, the videos were 97.3% effective in successfully conveying the intended magic tricks, and importantly, no participant reported the presence of a non-existent object in Videos 1, 2, or 4. All 420 participants generated one written report for each of the four videos they viewed, for a total 1260 separate verbal reports. Only 34 reports, from 27 separate participants, indicated that the trick was perceived as non-magical:

′ Twenty-one reports related to Video 1, the Miscellaneous Trick – four participants reported the correct method behind the Karate Coin Trick, 1 participant reported the correct method behind the Color Changing Silk Trick, and 16 participants erroneously stated that they saw the magician “throw” the chip upwards during the Levitating Poker Chip video (although this was not the genuine method, the trick was nevertheless perceived as non-magical);
′ Nine reports related to Video 2, the Vanish Trick – seven participants reported the correct method behind the Chip Vanish Trick, one participant reported the correct method behind the Silk Vanish Trick, and one participant reported the correct method behind the Crayon Vanish Trick;
′ Four reports related to Video 4, the Appearance Trick – four participants reported the correct method behind the Crayon Production.

#### Participants’ Written Reports for the Non-Magic Control (Video 3)

Video 3, the Non-Magic Control video, was not a conventional magic trick in that it was not designed to create an illusion of impossibility; instead, the magician performed an action that was intended to appear surprising but not to violate any natural or physical laws. As predicted, none of the participants reported seeing anything impossible or magical in the Non-Magic Control video, and importantly, no participant reported the presence of a non-existent object in Video 3. Some examples of the reports include: “He took a coin out of the cup and put it between his teeth” or “The man took the coin out of the cup and put it into his mouth. Then he waved his hands to the side, and rested his arms on the table afterward. Nothing magical happened.” The responses provided by the participants indicated that they were distinguishing between the magic trick videos (Videos 1, 2, and 4) and the Non-Magic Control (Video 3) because, unlike the reports for the magic trick videos, the participants did not report anything impossible or magical in response to Video 3.

#### Participants’ Written Reports for the Phantom Vanish Trick (Video 5)

Video 5, the Phantom Vanish Trick, was the critical video of the experiment. In contrast to the first four videos, no object was visible in this video; the Phantom Vanish Trick was intended to induce the illusory perception of a “phantom” object where no object was presented. Reports of phantom objects were categorized based on the participants’ written reports for Q1:

(1) Participants who only described the veridical events of the video were categorized as not having reported experiencing the PVI (e.g., “The magician pretended to take something out of the cup and make it disappear” or “His hands were empty. He reached into the cup. He then waved his hands around and then his hands remained empty”);
(2) Participants who reported that the magician took “something” out of the cup but did not provide any details about the object, were categorized as having reported experiencing the PVI but *not* reporting a specific object (e.g., “He took something out of the cup and it disappeared” or “The man takes the object from the cup into his hand. He makes a hand motion and it disappears. He points to his hand to show that it is indeed empty”);
(3) Participants who reported that the magician was performing with a specific object were categorized as having not only reported experiencing the PVI, but also having reported a specific object (e.g., “The magician removed a silver coin from the cup and placed it in his hand before making it disappear”).

In summary, of the 420 participants who responded to Q1 for Video 5, 284 participants (68%) were categorized as not having reported experiencing the PVI and 136 participants (32%) as having reported experiencing the PVI. Of the 136 participants categorized as having reported experiencing the PVI, 91 participants (21% of the total 420 participants) did not report a specific object and 45 participants (11% of the total 420 participants) reported a specific object. Of the 45 participants who reported specific objects, 39 (87%) reported seeing objects that were congruent with the objects they had been shown in the preceding videos. There were six exceptions, and all six participants reported seeing a coin (one participant in Object Condition 2, Red Ball; five participants in Object Condition 4, Silk Handkerchief).

### Participants’ Ratings for Surprising (Question 2), Impossible (Question 3), and Magical (Question 4) on the Spectators’ Experience Questionnaire

For every written report (Q1) collected for Videos 1–5, we also collected ratings from the participants for Surprising (Q2), Impossible (Q3), and Magical (Q4). See **Table 2** for the questions administered to the participants. These ratings (Q2–4) were included in the experimental design to corroborate the written reports for Q1.

#### Participants’ Ratings (Surprising, Impossible, and Magical) for the Magic Tricks (Videos 1, 2, and 4) Compared to the Non-Magic Control (Video 3)

**FIGURE 5:**
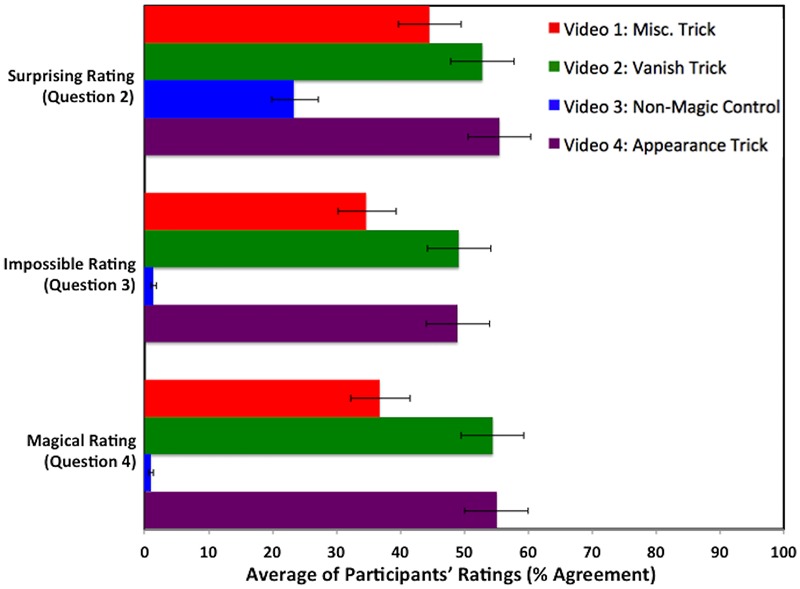
**Average of the participants’ ratings for Surprising, Impossible, and Magical on Videos 1–4.** Error bars represent 95% confidence intervals.

For Videos 1–4, the written reports (Q1) suggested that participants considered the Non-Magic Control (Video 3) to be less Impossible and Magical than the magic trick videos (Videos 1, 2, and 4). We used a linear mixed-effects model to compare participants’ ratings of Surprising (Q2), Impossible (Q3), and Magical (Q4) for the magic trick videos (Videos 1, 2, and 4) compared to the Non-Magic Control (Video 3). To fit the linear mixed-effects model, the error structure of the residuals need to be normal and heteroskadastic; satisfactory normality was achieved by applying a folded logarithmic transformation of the form: log((*x* + 1)/(101 – *x*)) to the ratings data. We treated pairings of videos and ratings as fixed effects, such that each of the four videos (Videos 1, 2, 3, and 4) was paired with each of the three ratings (Surprising, Impossible, and Magical) for a total of 12 fixed effects. Participants were treated as random effects. Models were fitted using the nlme package (Pinheiro et al., 2016) in R (R Core Team, 2016). See **Figure 5** for the participants’ ratings of Surprising, Impossible, and Magical for Videos 1–4.

Participants’ ratings for Surprising (Q2) were significantly lower for the Non-Magic Control Video 3 (*M* = 23.41, 95% CI [19.95, 27.25]) than for each of the magic trick videos: Video 1 (*M* = 44.54, 95% CI [39.65, 49.54], *t*(4609) = 8.23, *P* < 0.001); Video 2 (*M* = 52.85, 95% CI [46.84, 56.81], *t*(4609) = 11.09, *P* < 0.001); Video 4 (*M* = 55.53, 95% CI [50.53, 60.42], *t*(4609) = 12.02, *P* < 0.001).

Participants’ ratings for Impossible (Q3) were significantly lower for the Non-Magic Control Video 3 (*M* = 1.35, 95% CI [0.94, 1.85]) than for each of the magic trick videos: Video 1 (*M* = 34.66, 95% CI [30.24, 39.34], *t*(4609) = 27.33, *P* < 0.001); Video 2 (*M* = 49.18, 95% CI [44.18, 54.18], *t*(4609) = 32.48, *P* < 0.001); Video 4 (*M* = 48.97, 95% CI [43.98, 53.98], *t*(4609) = 32.41, *P* < 0.001).

Participants’ ratings for Magical (Q4) were significantly lower for the Non-Magic Control Video 3 (*M* = 0.99, 95% CI [0.64, 1.41]) than for each of the magic trick videos: Video 1 (*M* = 36.80, 95% CI [32.25, 41.59], *t*(4609) = 29.62, *P* < 0.001); Video 2 (*M* = 54.47, 95% CI [49.46, 59.39], *t*(4609) = 35.79, *P* < 0.001); Video 4 (*M* = 55.10, 95% CI [50.10, 60.00], *t*(4609) = 36.01, *P* < 0.001).

In summary, the ratings (Q2–4) corroborated the written reports for Q1, indicating that participants considered the Non-Magic Control (Video 3) to be less Surprising, Impossible, and Magical than the magic trick videos (Videos 1, 2, and 4). These findings for ratings Q2–4 further support the earlier findings for Q1, and demonstrate that participants were clearly distinguishing between the magic trick videos (Videos 1, 2, and 4) and the Non-Magic Control (Video 3).

#### Participants’ Ratings (Surprising, Impossible, and Magical) as Predicted by Participants’ Written Reports for the Phantom Vanish Trick (Video 5)

Participants’ written reports (Q1) for the Phantom Vanish Trick (Video 5) suggested that there were three different ways that participants responded to the PVI. We predicted that the Surprising (Q2), Impossible (Q3), and Magical (Q4) ratings from participants who were categorized as having reported experiencing the PVI (that is, participants who reported that they had seen an object apparently disappear during Video 5) would be higher than the ratings from participants who were categorized as not having reported experiencing the PVI (that is, participants, whose experience could be described simply as watching the magician pantomime an action without an object). We also predicted that the ratings from participants who were categorized as having reported experiencing the PVI and had also reported a specific object (e.g., a silver coin) would be higher than the ratings from participants who were categorized as having reported experiencing the PVI but had *not* reported a specific object (e.g., “the magician took something out of the cup”).

**FIGURE 6:**
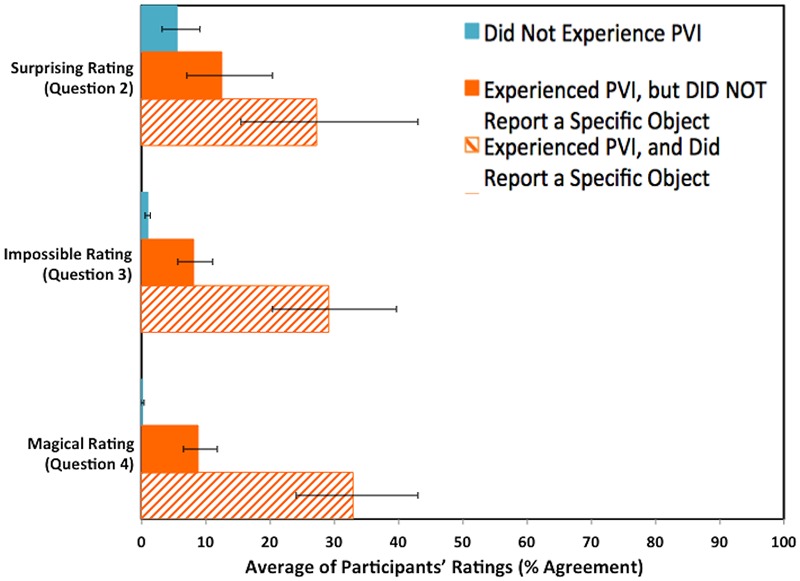
**Average of the participants’ ratings for Surprising, Impossible, and Magical on the Phantom Vanish Trick (Video 5) – a comparison of participants who did not report experiencing the Phantom Vanish Illusion (PVI) with participants who did report experiencing the PVI, and either did or did not report a specific object.** Error bars represent 95% confidence intervals.

We calculated three linear regression models to predict ratings of Surprising, Impossible, and Magical (respectively) from the participants’ written reports for Q1 of the Phantom Vanish Trick. To fit the three simple linear regression models, the error structure of the residuals need to be normal and heteroskadastic; satisfactory normality was achieved by applying a folded reciprocal transformation of the form: log((*x* + 1)/(101 – *x*)) to the ratings. For each model, our categorization of the participants’ reported experience of the PVI in Q1 for the Phantom Vanish Trick was used to predict the participants’ ratings of Surprising (Q2), Impossible (Q2), and Magical (Q2) for the Phantom Vanish Trick. Models were fitted using the lm package in R (R Core Team, 2016). See **Figure 6** for participants’ ratings for Surprising, Impossible, and Magical on the Phantom Vanish Trick (Video 5).

For each of the three models, we compared the simple regression model to a model that included four additional covariates. There were three categorical covariates: (1) participant gender (male or female); (2) computer screen-view setting (discrete or full-screen); (3) object used (i.e., Silver Coin, Red Ball, Poker Chip, Silk Handkerchief, or Crayon); and one continuous covariate: (4) participants’ self-reported interest in magic tricks (this covariate was transformed in the same way as the Surprising, Impossible, and Magical ratings, by applying a folded reciprocal transformation). The covariates were only included in the model reported if the likelihood test indicated that the covariates significantly improved the fit of the model. For example, none of the four covariates provided a significant improvement on the simple regression model for Impossible ratings, *F*_(7,410)_ = 1.89, *P* = 0.07 or for Magical ratings, *F*_(7,410)_ = 1.87, *P* = 0.07, and therefore the simple regression models are presented for these two ratings. In contrast, for Surprising ratings, the likelihood test indicated that the inclusion of two covariates – object used and participants’ self-reported interest in magic tricks – significantly improved the fit of the model, *F*_(8,412)_ = 0.39, *P* < 0.01, but that the inclusion of the two other covariates – participant gender and screen-view setting – did not improve the model, *F*_(2,410)_ = 0.39, *P* = 0.68.

##### Surprising Ratings for Video 5, the Phantom Vanish Trick

In the first of three linear regression models, we found that participants’ reported experience of the PVI (as categorized by their written responses to Q1 of Video 5) significantly predicted how Surprising they found the Phantom Vanish Trick (Q2, Video 5) while controlling for object used in the four videos that preceded the Phantom Vanish Trick (i.e., Silver Coin, Red Ball, Poker Chip, Silk Handkerchief, or Crayon) and the participants’ self-reported interest in magic tricks, *R*^2^ = 0.11, *F*_(7,412)_ = 7.59, *P <* 0.001. There was a significant difference between the Surprising ratings of participants who did not experience the PVI (*M* = 5.54, 95% CI [3.18, 9.09]) and participants who did experience the PVI but did not report a specific object (*M* = 12.44, 95% CI [7.11, 20.45]), *t*(412) = 3.29, *P <* 0.01, as well as between participants who did not experience the PVI and participants who did experience the PVI and did report a specific object (*M* = 27.24, 95% CI [15.50, 43.03]), *t*(412) = 5.35, *P <* 0.001. In addition, for participants who did experience the PVI, there was a significant difference in the Surprising ratings between participants who did and did not report a specific object, *t*(412) = 2.54, *P* = 0.02. This analysis supports our prediction that the participants’ written reports (Q1) for the Phantom Vanish Trick would be corroborated by their ratings of how Surprising (Q2) they found the Phantom Vanish Trick. Participants who we categorized (based on their written reports to Q1) as having reported experiencing the PVI rated the Phantom Vanish Trick as being more Surprising than those who we categorized as not having reported experiencing the PVI. Furthermore, participants who we categorized not only as having reported experiencing the PVI but also as having reported a specific object, rated the Phantom Vanish Trick as more Surprising than those who had not reported a specific object.

##### Impossible Ratings for Video 5, the Phantom Vanish Trick

In the second of three linear regression models, we found that participants’ reported experience of the PVI (as categorized by their written responses to Q1 of Video 5) significantly predicted how Impossible they found the Phantom Vanish Trick (Q3, Video 5), *R*^2^ = 0.31, *F*_(2,417)_ = 93.24, *P <* 0.001. There was a significant difference between the Impossible ratings of participants who did not experience the PVI (*M* = 0.98, 95% CI [0.65, 1.36]) and participants who did experience the PVI but did not report a specific object (*M* = 8.06, 95% CI [5.73, 11.09]), *t*(417) = 8.45, *P <* 0.001, as well as between participants who did not experience the PVI and participants who did experience the PVI and did report a specific object (*M* = 29.17, 95% CI [20.38, 39.75]), *t*(417) = 12.01, *P <* 0.001. In addition, for participants who did experience the PVI, there was a significant difference in the Impossible ratings between participants who did and did not report a specific object, *t*(417) = 5.10, *P* < 0.001. This analysis supports our prediction that the participants’ written reports (Q1) for the Phantom Vanish Trick would be corroborated by their ratings of how Impossible (Q3) they found the Phantom Vanish Trick. Participants who we categorized (based on their written reports to Q1) as having reported experiencing the PVI rated the Phantom Vanish Trick as being more Impossible than those who we categorized as not having reported experiencing the PVI. Furthermore, participants who we categorized not only as having reported experiencing the PVI but also as having reported a specific object, rated the Phantom Vanish Trick as more Impossible than those who had not reported a specific object.

##### Magical Ratings for Video 5, the Phantom Vanish Trick

In the third of three linear regression models, we found that participants’ reported experience of the PVI (as categorized by their written responses to Q1 of Video 5) significantly predicted how Magical they found the Phantom Vanish Trick (Q4, Video 5), *R*^2^ = 0.37, *F*_(2,417)_ = 127.5, *P <* 0.001. There was a significant difference between the Magical ratings of participants who did not experience the PVI (*M* = 0.89, 95% CI [0.60, 1.22]) and participants who did experience the PVI but did not report a specific object (*M* = 8.91, 95% CI [6.56, 11.90]), *t*(417) = 10.01, *P <* 0.001, as well as between participants who did not experience the PVI and participants who did experience the PVI and did report a specific object (*M* = 32.93, 95% CI [24.09, 43.11]), *t*(417) = 14.07, *P <* 0.001. In addition, for participants who did experience the PVI, there was a significant difference in the Magical ratings between participants who did and did not report a specific object, *t*(417) = 5.81, *P <* 0.001. This analysis supports our prediction that the participants’ written reports (Q1) for the Phantom Vanish Trick would be corroborated by their ratings of how Magical (Q4) they found the Phantom Vanish Trick. Participants who we categorized (based on their written reports to Q1) as having reported experiencing the PVI rated the Phantom Vanish Trick as being more Magical than those who we categorized as not having reported experiencing the PVI. Furthermore, participants who we categorized not only as having reported experiencing the PVI but also as having reported a specific object, rated the Phantom Vanish Trick as more Magical than those who did not report a specific object.

## Discussion

Our experiment investigated the illusory presence of objects in scenes where no object was presented. The PVI demonstrates that spectators’ expectations, in response to magic tricks, can lead them to imagine the existence of an object that “ought to be there.” In some cases, this imagined representation was vivid enough to be mistaken for a veridical visual perception. Thus, this experiment extends previous research demonstrating that magicians’ misdirection techniques can induce misperceptions of visual experiences.

One-third of our participants reported having been shown an object after watching a video where no object was presented. Our PVI paradigm is the first investigation of sleight-of-hand magic tricks that has involved participants spontaneously reporting their illusory experiences. After watching each video, participants provided written reports describing what they had been shown. In addition to collecting written reports, we asked the participants to rate how surprising, impossible, and magical they considered the videos. These ratings served to corroborate the written reports: participants who reported phantom objects rated the Phantom Vanish Trick video to be more surprising, impossible, and magical than those who did not experience the illusion. Past research, on false transfer tricks (e.g., Cui et al., 2011; Beth and Ekroll, 2015) and on the Vanishing Ball Illusion (e.g., Triplett, 1900; Kuhn and Land, 2006; Thomas and Didierjean, 2016a), has involved misleading participants about the motion and location of an object: the object was shown, and then was apparently passed from one hand to the other while secretly being retained in the first hand; or, the object was shown and then apparently tossed into the air while being secretly retained in the hand (or secretly dropped into the magician’s lap). In contrast, the PVI paradigm entirely eliminates the need to present an object during the critical trial. Overall, our paradigm provides strong evidence that participants who were categorized as having experienced the illusion were honestly confusing “phantom” objects for genuine objects. Our results also suggest that the participants’ reports of “phantom” objects cannot be attributed to demand characteristics. Participants’ responses to the Spectators’ Experience Questionnaire for Video 3 (the Non-Magic Control video) indicated that the participants were not simply describing every video they watched as being impossible or magical merely because they had been told that they would be watching magic tricks. No participant reported seeing anything impossible or magical after watching Video 3, which was rated as significantly less impossible and less magical than the magic trick videos (Videos 1, 2, and 4). These results also raise intriguing questions about exactly what makes-up these “phantom” objects, and what these reports reveal about human perception.

One might argue that the participants’ reports of illusory objects can be attributed to memory errors rather than perceptual errors. In other words, participants who reported seeing the phantom objects may not have had a phenomenological experience of “seeing” the object during the Phantom Vanish Trick video, instead they may have retrospectively confabulated the object after they had been cued to describe the events in the video. The design of our experiment allows us to exclude two memory-related factors that might otherwise have contributed to the illusion: post-event misinformation (including verbal and non-verbal information) and false verbal suggestions.

There is a rich literature on misinformation and the unreliability of eye-witness testimony. Researchers have repeatedly demonstrated that people are capable of confusing imaginary events with real memories (see Loftus, 2005 for a review). The idea that people can be led to report imaginary events has been established by research on the effects of leading questions. Loftus and Palmer (1974) showed that participants could be induced to remember seeing things that were not presented in response to leading questions. One week after having watched a video of a car accident, participants were explicitly asked: “Did you see any broken glass?” The reported false memories of broken glass could not have been derived directly from the video, because the video did not actually show any broken glass; thus, the false memory was arguably induced by the question itself. Other researchers have demonstrated that false verbal suggestions presented *co-currently* with events can also induce false reports (Wiseman et al., 2003; Wiseman and Greening, 2005; Wilson and French, 2014).

Similar results have been obtained in the absence of verbal misinformation, such as when Gurney et al. (2013) demonstrated that participants who were being questioned about a video recording of a robbery could be induced to report false information in response to non-verbal “leading gestures.” For example, when the interviewer stroked his chin, while asking participants if they noticed any distinguishing features on the robber in the video, participants were more likely to report falsely that the robber had a beard compared to participants who were asked the question without the accompanying gesture.

In both our PVI paradigm, and previous research with the Vanishing Ball Illusion paradigm, the silent video clips that serve as stimuli preclude the use of false verbal suggestions during stimulus presentation. The Vanishing Ball Illusion paradigm involves asking participants a series of questions relating to the ball. After watching the video of the trick, the participants were asked to mark the location of the last place they saw the ball on a still picture that depicted the magician. Participants were considered to be sensitive to the illusion if they indicated that they had seen the ball leave the magician’s hand on the last throw. They were considered insensitive to the illusion if they (correctly) marked the magician’s hand as being the last place where they had seen the ball. Participants were then asked to describe what they saw, asked how the illusion was created, and given a yes/no forced choice question: “Did you see the ball move up on the final throw?” (Kuhn and Land, 2006). In contrast, in our PVI paradigm, the participants freely reported seeing the phantom object in response to a question that asked them to recall “actions” and “events” but made no specific reference to an object. In the PVI paradigm, given that there was no object presented during the Phantom Vanish Trick video, care was taken to ask participants non-leading questions, so as to rule-out the potential for post-event information to generate introspective errors during the participants’ recollection of the events. The omission of a direct question about the object in the PVI paradigm may partially account for the fact that 68% of our 420 participants did not report experiencing the PVI.

With regards to ecological versus inferential theories of perception, our results do not support Gibson’s (1982) specific ecological prediction that healthy sober people can never “see” a non-existent object – 32% of the 420 participants who completed our experiment reported that they had been shown objects when none had been presented. These results support a more inferential model of human perception. This concept, that conscious phenomenological experience is actively constructed by combining top-down cognitive processes with bottom-up sensory information, may offer insight into how participants came to experience the PVI.

Gregory’s (2009) framework for classifying illusory pheno menon includes both paradoxical illusions and fictional illusions. Paradoxical illusions refer to perceptions that seem to be logically impossible (e.g., Kulpa, 1987), while fictional illusions refer to perceptual experiences that fail to directly correspond with sensory information (e.g., modal and amodal completions). Fictional illusions do not necessarily need to be based on false assumptions. For example, the amodal completion of objects is often based on accurate inferences: if one were to see a person standing behind a picket fence, and this caused the image of the person to be partially occluded, it would normally be correct to assume that the person’s body really extends to areas occluded by the fence, rather than them being neatly sliced into separate sections.

We propose that sleight-of-hand illusions be classified as “paradoxical fictions.” Magic tricks are designed to exploit spectators’ inferences, along with their intuitions about their own perceptual systems, to create the “illusion of impossibility” (e.g., Nelms, 1969; Ortiz, 2006). Magic tricks are paradoxical in that an effective magic trick will appear to violate the laws of nature. For example, in a “vanishing” trick, an object appears to pass from existence into non-existence. Magic tricks are fictional in that the spectators’ perceptual experiences can often differ dramatically from bottom-up sensory information, as in the case with our PVI or with the Vanishing Ball Illusion. These magical experiences can be considered “failures of visual metacognition” (Beth and Ekroll, 2015, p. 520). That is to say, we tend to believe what we see, and we are generally unaware of the discrepancy between how our perceptual system actually works and how we think it ought to work. Magic effects result from “hacking” otherwise adaptive perceptual processes to create false fictional experiences that lead to paradoxical experiences. In the case of the PVI, people would generally not believe that they could “see” an object where one does not exist. The “illusion of impossibility” occurs when the magician reveals the conflict between reality and the spectators’ perceptual experience. At the “climax” of the Phantom Vanish Trick, the magician clearly shows that both of his hands are empty. Because the spectator does not believe that they could have misperceived an object that was never really there, they are unable to intuit that the true method is even possible.

One explanation for why participants reported phantom objects during the Phantom Vanish Trick is that the participants’ top-down expectations about the object outweighed the bottom-up sensory counter-evidence (the absence of the object; Kuhn and Rensink, 2016). Various top-down expectations may have contributed to the creation of an amodal spatiotemporal representation of the object (Beth and Ekroll, 2015; Thomas and Didierjean, 2016b). Among the 136 participants who were categorized as having experienced the PVI, those who reported a specific object (e.g., a coin) might have based their reports on the perceptual experience of modal completion (they had the impression that an object had been openly displayed), while those who reported an object but did not specify which object, might have based their reports on an amodal completion (they had the impression that an object was presented, but that it was occluded by the magician’s hand). However, one limitation of our written response format for Question 1, in which participants freely reported their experiences, is that we cannot determine whether the participants who did not report a specific object might have been capable of naming a specific object, if asked. In any case, all participants who reported having seen a phantom object apparently committed a metacognitive error of failing to distinguish the representation from a real object.

Participants’ top-down expectations may have been influenced by multiple factors. Because there is no object presented during the critical video, the PVI paradigm can potentially be used to isolate a variety of variables that may contribute to sleight-of-hand illusions, including perceptual priming (i.e., the expectations established by the preceding videos^4^), social cues (i.e., the gaze and head direction of the magician), and the convincingness of the magician’s pantomime (i.e., the grasp of the non-existent object). In future studies, each of these factors could be manipulated to isolate their respective roles in creating the PVI. The preceding four videos in the five-video sequence did include real objects. These videos may have served as perceptual primes, analogous to the real tosses that precede the false throw in the Vanishing Ball Illusion. One experiment (Kuhn and Rensink, 2016) has shown that manipulating the perceptual priming aspect of a magic trick (the real tosses that precede the false throw in the Vanishing Ball Illusion paradigm) affects the probability that participants will experience the illusion, and that the illusion can still be effective when the perceptual primes are eliminated entirely from the trick (i.e., the magician simply showed the ball and then immediately performed the false throw without making any real tosses). This suggests that our PVI might still be effective for some participants, even if the experiment were modified to reduce or even eliminate the preceding videos. For example, one could manipulate which objects are shown in the preceding videos, or manipulate the number of videos that precede the Phantom Vanish Trick. Additionally, the social cues of the magician could be manipulated by occluding the magician’s face, or by including a condition where the magician maintains a fixed, unmoving gaze (see Thomas and Didierjean, 2016a).

In summary, the PVI represents a new contribution to the rapidly growing field of the “Science of Magic” – the use of methodologies inspired by performance magic to experimentally investigate human psychology. Just as optical illusions and visual arts represent a resource for visual scientists, the more elaborate illusions created by magic performances can be used to examine more complex elements of human visual cognition. We hope that the PVI paradigm represents not only a novel contribution to the Science of Magic, but more generally, a new tool for perception researchers looking to untangle the complex influences of top-down factors on the way people process dynamic visual scenes.

## Author Contributions

MT designed the experiment, collected and analyzed the data, drafted and revised the manuscript. AA designed the experiment, analyzed the data, drafted and revised the manuscript. AW assisted with the experimental design, collected the data, and revised the manuscript.

## Conflict of Interest Statement

The authors declare that the research was conducted in the absence of any commercial or financial relationships that could be construed as a potential conflict of interest.

## Footnotes

1 A consideration here is that data collection was done online as opposed to in a more traditional laboratory setting. A popular argument for not conducting research online is that the data collected is for some reason unreliable; for example, because it is unknown if participants are properly paying attention. One way of assessing this is to ask participants a “catch” question at the end of the study (e.g., “do not click continue, rather click the small circle at the bottom of the screen”; Oppenheimer et al., 2009). Hauser and Schwarz (2016) tested this issue and found that, while Mechanical Turk participants failed such a task 5% of the time, a staggering 61% of laboratory-based participants also failed the task. We did not include such a catch question for this reason. In terms of the overall data reliability issue, almost all attempts of replicating laboratory-based psychology studies online have been successful (e.g., Germine et al., 2012; Crump et al., 2013; Klein et al., 2014), with the few exceptions being attributable to inconsistencies in the hardware used by the participants. Hardware discrepancies can make it difficult to present very short duration stimuli onscreen accurately, such as is important in the masked-priming study, which failed to be completely replicated (Crump et al., 2013). Our stimuli were videos, all of which were over 10 seconds long, and so would not thus be affected. Of course, we also benefited from our online participants being less WEIRD (Western, Educated, Industrialized, Rich, and Democratic; Henrich et al., 2010; Berinsky et al., 2012) than laboratory-based participants. (Although some would argue that online participants are weird in their own right, for example, by being much more computer literate than individuals recruited off the street; for a discussion on this and a more in depth overview of the above issues, the reader is directed to Woods et al., 2015.)

2 www.xperiment.mobi

3 https://www.youtube.com/playlist?list=PLnfidBe0mwswzhAjRLRLMDrEO0zGJYi23

4 Of interest here is the fact that six participants in our experiment did *not* actually report seeing a phantom object that was congruent with the object they had been shown in previous videos. This might be attributable to the fact that the magician depicted in the videos predominately practices and performs slight-of-hand magic with coins, meaning that his pantomimed grasp shown during Video 5 (Phantom Vanish Trick) may have been most closely related to the grasp that would be used to hold a coin. Alternatively, participants might have had a prior expectation established outside of the experiment, that magic performances often involve disappearing coins.

## References

[B1] Barnhart A. S. (2010). The exploitation of gestalt principles by magicians. *Perception* 39 1286–1289. 10.1068/p6766 21125955

[B2] Barnhart A. S. Goldinger S. D. (2014). Blinded by magic: eye-movements reveal the misdirection of attention. *Front. Psychol.* 5:1461 10.3389/fpsyg.2014.01461 PMC4269107 25566139

[B3] Berinsky A. J. Huber G. A. Lenz G. S. (2012). Evaluating online labor markets for experimental research: Amazon.com’s mechanical turk. *Polit. Anal.* 21 351–368. 10.1093/pan/mpr057

[B4] Beth T. Ekroll V. (2015). The curious influence of timing on the magical experience evoked by conjuring tricks involving false transfer: decay of amodal object permanence? *Psychol. Res.* 79 513–522. 10.1007/s00426-014-0584-2 24941913

[B5] Binet A. (1896). *Psychology of Prestidigitation.* Annual Report of the Board of Regents of the Smithsonian Institution. Washington, DC: Government Printing Office.

[B6] Bobo J. B. (1952). *Modern Coin Magic.* Minneapolis, MN: C.W. Jones.

[B7] Cavina-Pratesi C. Kuhn G. Ietswaart M. Milner A. D. (2011). The magic grasp: motor expertise in deception. *PLoS ONE* 6:e16568 10.1371/journal.pone.0016568 PMC3036651 21347416

[B8] Christopher M. Christopher M. B. (1973). *The Illustrated History of Magic.* New York, NY: Crowell.

[B9] Crump J. C. McDonnel J. V. Gureckis T. M. (2013). Evaluating Amazon’s mechanical turk as a tool for experimental behavioural research. *PLoS ONE* 8:e57410 10.1371/journal.pone.0057410 PMC3596391 23516406

[B10] Cui J. Otero-Millan J. Macknik S. L. King M. Martinez-Conde S. (2011). Social misdirection fails to enhance a magic illusion. *Front. Hum. Neurosci.* 5:103 10.3389/fnhum.2011.00103 PMC3202226 22046155

[B11] Dessoir M. (1893). The psychology of legerdemain. *Open Court* 12 3599–3606.

[B12] Ekroll V. Sayim B. Wagemans J. (2013). Against better knowledge: the magical force of amodal volume completion. *Iperception* 4 511–515. 10.1068/i0622sas 25165509 PMC4129385

[B13] Germine L. Nakayama K. Duchaine B. C. Chabris C. F. Chatterjee G. Wilmer J. B. (2012). Is the Web as good as the lab? Comparable performance from Web and lab in cognitive/perceptual experiments. *Psychon. Bull. Rev.* 19 847–857. 10.3758/s13423-012-0296-9 22829343

[B14] Gibson J. J. (1982). “Ecological physics, magic, and reality,” in *Reasons for Realism: Selected Essays of James J. Gibson* eds Gibson J. J. Reed E. S. Jones R. (Mahwah, NJ: Lawrence Erlbaum Associates).

[B15] Gregory R. (1982). Conjuring. *Perception* 11 631–633.

[B16] Gregory R. L. (1997). Knowledge in perception and illusion. *Philos. Trans. R. Soc. Lond. B Biol. Sci.* 352 1121–1127. 10.1098/rstb.1997.0095 9304679 PMC1692018

[B17] Gregory R. L. (2009). *Seeing Through Illusions.* Oxford: Oxford University Press.

[B18] Gurney D. J. Pine K. J. Wiseman R. (2013). The gestural misinformation effect: skewing eyewitness testimony through gesture. *Am. J. Psychol.* 126 301–314. 10.5406/amerjpsyc.126.3.0301 24027944

[B19] Hauser D. J. Schwarz N. (2016). Attentive Turkers: MTurk participants perform better on online attention checks than do subject pool participants. *Behav. Res. Methods* 48 400–407. 10.3758/s13428-015-0578-z 25761395

[B20] Helmholtz H. (1867). “Concerning the perceptions in general,” in *Treatise on Physiological Optics* 3rd Edn Vol. III trans. J. P. C. Southall 1925 (New York, NY: Dover).

[B21] Henrich J. Heine S. J. Norenzayan A. (2010). The weirdest people in the world? *Behav. Brain Sci.* 33 61–135. 10.1017/S0140525X0999152X 20550733

[B22] Hodgson R. Davey S. (1887). The possibilities of mal-observation and lapse of memory from a practical point of view. *Proc. Soc. Psych. Res.* 4 381–495.

[B23] Houdin R. (1868/1881). *The Secrets of Stage Conjuring (trans. Professor Hoffman [pseudonym for Lewis, A. J.]).* London: George Routledge and Sons.

[B24] Hyman R. (1989). The psychology of deception. *Annu. Rev. Psychol.* 40 133–154. 10.1146/annurev.ps.40.020189.001025

[B25] Jastrow J. (1888). The psychology of deception. *Pop. Sci. Mon.* 34 145–157.

[B26] Jastrow J. (1896). Psychological notes upon sleight-of-hand experts. *Science* 3 685–689. 10.1126/science.3.71.685 17743833

[B27] Johansson P. Hall L. Sikstrom S. Olsson A. (2005). Failure to detect mismatches between intention and outcome in a simple decision task. *Science* 310 116–119. 10.1126/science.1111709 16210542

[B28] Klein R. A. Ratliff K. A. Vianello M. Adams R. B. Jr. Bahník Š Bernstein M. J. (2014). Investigating variation in replicability. *Soc. Psychol.* 45 142–152.

[B29] Kuhn G. Amlani A. A. Rensink R. A. (2008). Towards a science of magic. *Trends Cogn. Sci.* 12 349–354. 10.1016/j.tics.2008.05.008 18693130

[B30] Kuhn G. Caffaratti H. A. Teszka R. Rensink R. A. (2014). A psychologically-based taxonomy of misdirection. *Front. Psychol.* 5:1392 10.3389/fpsyg.2014.01392 PMC4260479 25538648

[B31] Kuhn G. Findlay J. M. (2010). Misdirection, attention and awareness: inattentional blindness reveals temporal relationship between eye movements and visual awareness. *Q. J. Exp. Psychol.* 63 136–146. 10.1080/17470210902846757 19459083

[B32] Kuhn G. Land M. F. (2006). There’s more to magic than meets the eye. *Curr. Biol.* 16 950–951. 10.1016/j.cub.2006.10.012 17113372

[B33] Kuhn G. Rensink R. A. (2016). The vanishing ball illusion: a new perspective on the perception of dynamic events. *Cognition* 148 64–70. 10.1016/j.cognition.2015.12.003 26735583

[B34] Kuhn G. Tatler B. W. (2005). Magic and fixation: now you don’t see it, now you do. *Perception* 34 1155–1161. 10.1068/p3409bn1 16245492

[B35] Kuhn G. Teszka R. Tenaw N. Kingstone A. (2016). Don’t be fooled! Attentional responses to social cues in a face-to-face and video magic trick reveals greater top-down control for overt than covert attention. *Cognition* 146 136–142. 10.1016/j.cognition.2015.08.005 26407341

[B36] Kulpa Z. (1987). Putting order in the impossible. *Perception* 16 201–214. 10.1068/p160201 3684482

[B37] Lamont P. (2010). The misdirected quest. *Psychologist* 23 978–980.

[B38] Lamont P. (2015). Problems with the mapping of magic tricks. *Front. Psychol.* 6:855 10.3389/fpsyg.2015.00855 PMC4477052 26157408

[B39] Lamont P. Wiseman R. (1999). *Magic in Theory: An Introduction to the Theoretical and Psychological Elements of Conjuring.* Hertfordshire: University of Hertfordshire Press.

[B40] Loftus E. F. (2005). Planting misinformation in the human mind: a 30-year investigation of the malleability of memory. *Learn. Mem.* 12 361–366. 10.1101/lm.94705 16027179

[B41] Loftus E. F. Palmer J. C. (1974). Reconstruction of automobile destruction: an example of the interaction between language and memory. *J. Verbal Learn. Verbal Behav.* 13 585–589. 10.1016/S0022-5371(74)80011-3

[B42] Macknik S. L. King M. Randi J. Robbins A. Thompson J. Martinez-Conde S. (2008). Attention and awareness in stage magic: turning tricks into research. *Nat. Rev. Neurosci.* 9 871–879. 10.1038/nrn2473 18949833

[B43] Maskelyne J. N. Devant D. (1911). *Our Magic.* London: George Routledge and Sons.

[B44] Memmert D. (2010). The gap between inattentional blindness and attentional misdirection. *Conscious. Cogn.* 19 1097–1101. 10.1016/j.concog.2010.01.001 20122850

[B45] Nanay B. (2009). “Four theories of amodal perception,” in *Proceedings of the 29th Annual Conference of the Cognitive Science Society (CogSci 2007)* eds McNamara D. S. Trafton J. G. (Hillsdale, NJ: Erlbaum) 1331–1336.

[B46] Nelms H. (1969). *Magic and Showmanship: A Handbook for Conjurers.* North Chelmsford, MA: Courier Corporation.

[B47] Oppenheimer D. M. Meyvis T. Davidenko N. (2009). Instructional manipulation checks: detecting satisficing to increase statistical power. *J. Exp. Soc. Psychol.* 45 867–872. 10.1016/j.jesp.2009.03.009

[B48] Ortiz D. (2006). *Designing Miracles: Creating the Illusion of Impossibility.* El Dorado Hills, CA: A-1 Magical Media.

[B49] Phillips F. Natter M. B. Egan E. J. (2015). Magically deceptive biological motion—the french drop sleight. *Front. Psychol.* 6:371 10.3389/fpsyg.2015.00371 PMC4391225 25914654

[B50] Pinheiro J. Bates D. DebRoy S. Sarkar D. R Core Team (2016). *nlme: Linear and Nonlinear Mixed Effects Models. R Package Version 3.1-126.* Available at: http://CRAN.R-project.org/package=nlme

[B51] R Core Team (2016). *R: A Language and Environment for Statistical Computing.* Vienna: R Foundation for Statistical Computing. Available at: http://www.R-project.org/

[B52] Reips U. Funke F. (2008). Interval-level measurement with visual analogue scales in internet-based research: VAS generator. *Behav. Res. Methods* 40 699–704. 10.3758/BRM.40.3.699 18697664

[B53] Rensink R. A. Kuhn G. (2015). A framework for using magic to study the mind. *Front. Psychol.* 5:1508 10.3389/fpsyg.2014.01508 PMC4313584 25698983

[B54] Rissanen O. Pitkänen P. Juvonen A. Kuhn G. Hakkarainen K. (2014). Expertise among professional magicians: an interview study. *Front. Psychol.* 5:1484 10.3389/fpsyg.2014.01484 PMC4274899 25566156

[B55] Scot R. (1584). *The Discoverie of Witchcraft, Wherein the Lewde Dealing of Witches and Witchmongers is Notablie Detected, the Knaverie of Conjurors, the Impietie of Inchantors, the Follie of Soothsaiers, the Impudent Falsehood of Cousenors, the Infidelitie of Atheists, the Pestilent Practices of Pythonist, the Curiositie of Figurecasters, the Vanitie of Dreamers, the Beggerlie Art of Alcumystrie, The Abhomination of Idolatrie, the Horrible art of Poisoning, the Vertue and Power of Natural Magike, and all the Conveiances of Legierdemaine and Juggling are Deciphered: and Many other Things Opened, which have Long Lien Hidden, Howbeit Verie Necessarie to be Knowne. Heereunto is added a Treatise upon the Nature and Substance of Spirits and Divels, &c.* London: William Brome.

[B56] Shephard S. (1946). The pretense coin vanish. *Magicana* 1 8.

[B57] Smith T. J. Lamont P. Henderson J. M. (2012). The penny drops: change blindness at fixation. *Perception* 41 489–492. 10.1068/p7092 22896921

[B58] Smith T. J. Lamont P. Henderson J. M. (2013). Change blindness in a dynamic scene due to endogenous override of exogenous attentional cues. *Perception* 42 884–886. 10.1068/p7377 24303751

[B59] Tachibana R. Kawabata H. (2014). The effects of social misdirection on magic tricks: how deceived and undeceived groups differ. *Iperception* 5 143–146. 10.1068/i0640sas 25469219 PMC4249983

[B60] Thomas C. Didierjean A. (2016a). No need for a social cue! A masked magician can also trick the audience in the vanishing ball illusion. *Attent. Percept. Psychophys.* 78 21–29. 10.3758/s13414-015-1036-9 26676869

[B61] Thomas C. Didierjean A. (2016b). The ball vanishes in the air: can we blame representational momentum? *Psychon. Bull. Rev.* 10.3758/s13423-016-1037-2 [Epub ahead of print]. 27184252

[B62] Thomas C. Didierjean A. Maquestiaux F. Gygax P. (2015). Does magic offer a cryptozoology ground for psychology? *Rev. Gen. Psychol.* 19 117–128. 10.1037/gpr0000041

[B63] Thomas C. Didierjean A. Nicolas S. (2016). Scientific study of magic: Binet’s pioneering approach based on observation and chronophotography. *Am. J. Psychol.* 129 315–328. 10.5406/amerjpsyc.129.3.0313 29558594

[B64] Triplett N. (1900). The psychology of conjuring deceptions. *Am. J. Psychol.* 11 439–510. 10.1016/j.tics.2016.04.006

[B65] Whaley B. (2006). *Detecting Deception: A Bibliography of Counterdeception Across Time, Cultures, and Disciplines* 2nd Edn. Washington, DC: Foreign Denial & Deception Committee, National Intelligence Council.

[B66] Wilson K. French C. C. (2014). Magic and memory: using conjuring to explore the effects of suggestion, social influence, and paranormal belief on eyewitness testimony for an ostensibly paranormal event. *Front. Psychol.* 5:1289 10.3389/fpsyg.2014.01289 PMC4230037 25431565

[B67] Wiseman R. Greening E. (2005). It’s still bending: verbal suggestion and alleged psychokinetic ability. *Br. J. Psychol.* 96 115–127. 10.1348/000712604X15428 15826327

[B68] Wiseman R. Greening E. Smith M. (2003). Belief in the paranormal and suggestion in the seance room. *Br. J. Psychol.* 94 285–297. 10.1348/000712603767876235 14511544

[B69] Woods A. T. Velasco C. Levitan C. A. Wan X. Spence C. (2015). Conducting perception research over the Internet: a tutorial review. *PeerJ* 3:e1058 10.7717/peerj.1058 PMC4517966 26244107

[B70] Wundt W. (1879). Spiritualism as a scientific question. *Pop. Sci. Mon.* 15 577–593.

[B71] Zöllner J. C. (1878). On space of four dimensions. *Q. J. Sci.* 8 227–237.

